# Noble metal-modified octahedral anatase titania particles with enhanced activity for decomposition of chemical and microbiological pollutants

**DOI:** 10.1016/j.cej.2016.05.138

**Published:** 2017-06-15

**Authors:** Z. Wei, M. Endo, K. Wang, E. Charbit, A. Markowska-Szczupak, B. Ohtani, E. Kowalska

**Affiliations:** a Institute for Catalysis, Hokkaido University, N21, W10, 001-0021 Sapporo, Japan; b Institute of Chemical and Environmental Engineering, West Pomeranian University of Technology in Szczecin, ul. Pulaskiego 10, 70-322 Szczecin, Poland

**Keywords:** Antimicrobial activity, Modified titania, Octahedral anatase particles, Plasmonic nanoparticles, Photocatalytic activity, Visible light-responsive photocatalysts

## Abstract

Octahedral anatase particles (OAPs) were prepared by hydrothermal (HT) reaction of titanate nanowires (TNWs). OAPs were modified with noble metals (Au, Ag, Cu and Pt) by two photodeposition methods: in the absence and in the initial presence of oxygen in the system. Photocatalytic activities for oxidative decomposition of acetic acid and anaerobic dehydrogenation of methanol under UV/vis irradiation and for oxidation of 2-propanol under visible light irradiation were investigated. Antibacterial activities for bacteria (*Escherichia coli*) and fungi (*Candida albicans*) were investigated in the dark and under UV irradiation and/or visible light irradiation. It was found that the kind of metal deposition significantly influenced the properties of photocatalysts obtained and thus their photocatalytic and antimicrobial activities. Modification of OAPs with metallic deposits resulted in enhanced photocatalytic activities for all tested systems. Pt-modified OAPs showed the highest activity for dehydrogenation of methanol due to their highest work function and lowest activation overpotential of hydrogen evolution. Cu-modified OAPs exhibited the highest activity for oxidative decomposition of acetic acid under UV/vis irradiation, probably due to the heterojunction between Cu oxides and TiO_2_. On the other hand, Au-modified OAPs showed the highest photocatalytic activity under visible light irradiation due to their plasmonic properties. Bare OAPs, prepared with various durations of the HT reaction, did not have any antibacterial properties in the dark, while their activity under UV/vis irradiation was correlated with their photocatalytic activities for dehydrogenation of methanol and decomposition of acetic acid. Antimicrobial activity of modified OAPs in the dark and under visible light irradiation was the highest for Ag-modified OAPs. Under UV irradiation, Cu-modified OAPs showed the highest activity for inactivation of both bacteria and fungi.

## Highlights

• Enhancement of titania activity by modification with Au, Pt, Ag and Cu.
• Significant change of properties by metal photodeposition in the presence of oxygen.
• Correlation of antimicrobial and photocatalytic properties for octahedral anatase.
• Zero-valent Pt and Au and positively charged Cu and Ag in the ambient environment.
• Visible activity of Au/OAPs and Ag/OAPs, due to activation of plasmon resonance.

## Graphical abstract

**Figure:**
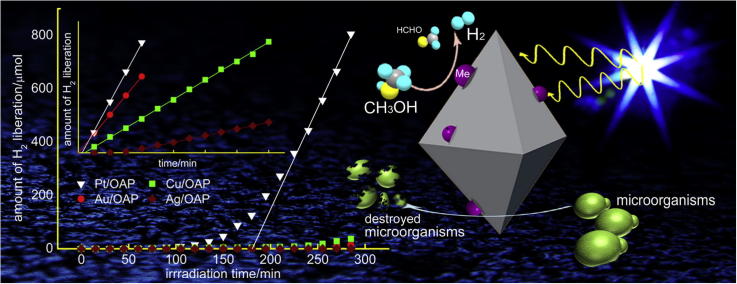

## 1 Introduction

An enormous amount and various kinds of indoor and outdoor pollutants (both chemical and microbiological) that have adverse effects on health are released into environment every day. Although environmental contamination by chemical pollutants is usually well-monitored and controlled [1], [2], [3], microbiological pollution is often accidental and results in uncontrolled spread of microorganisms. Microorganisms can grow rapidly without continuous release into the environment. For example, some bacteria and fungi can rapidly reproduce on the surfaces of building materials such as wood, paper, gypsum, ceramics, minerals, paints and plastics [4]. Moreover, positive, negative or neutral interactions between various bacteria and fungi *via* infochemicals produced by them [5], [6], as well as between microorganisms and chemical pollutants, may have a crucial impact on the environment. Interactions between microorganisms, chemical compounds and the environment are complex and unpredictable. Therefore, the development of environmentally friendly techniques that can efficiently degrade pollutants is needed.

Photocatalysis is considered to be one of the best methods for environmental purification since additional chemical compounds such as oxidants (ozone, hydrogen peroxide and chlorine) [7], [8], [9], [10], [11], [12], [13], [14] are not introduced into the environment [15], [16]. Energy consumption is also much less than that in other advanced oxidation processes (AOPs) such as wet air oxidation [17], supercritical water oxidation [18] and H_2_O_2_/UV-C [19] because UV-A lamps and even free solar radiation can be used for photocatalyst activation [20].

Among the various heterogeneous photocatalysts, titanium(IV) oxide (titania, TiO_2_) is one of the most widely used due to its advantages such as good stability, strong redox ability, relative nontoxicity, low cost and high availability [21], [22], [23], [24]. However, recombination of charge carriers (a typical phenomenon for all semiconducting materials) and inability to absorb visible light due to its wide band-gap (ca. 3.0 eV for rutile and 3.2 eV for anatase) limit its application to regions of the world with high intensity of solar radiation. Many studies have therefore been carried out to improve the performance of titania by its surface modification, doping and preparation of coupled nanostructures [25], [26], [27], [28], [29], [30], [31], [32], [33], [34]. Modification with metals (metallic nanoparticles (NPs) and clusters [35], [36], [37], [38], [39], metal complexes [40], [41]) has been one of the most frequently used methods for both enhancement of activity under UV light and activation of titania by visible light irradiation. Metallic deposits under UV irradiation work as an electron sink that inhibits the recombination of charge carriers. Under visible light irradiation, either narrowing of the band-gap [42], [43], [44] or energy/electron transfer from the modifier to titania [45], [46], [47] causes a visible light response. Recently, modification of titania with NPs of noble metals that have a visible light absorption property due to localized surface plasmon resonance (LSPR) has been extensively studied, and such materials obtained are called plasmonic photocatalysts. Although there have been controversial results of studies regarding the mechanism of their action, i.e., transfer of energy [48], transfer of charge carriers [49], [50] and plasmonic heating [51], their photocatalytic activity in a broad range of irradiation conditions makes them promising materials for environmental purification and energy conversion. In addition, antibacterial properties of silver and copper extend their possible application to dark conditions. It is thought that the mechanism of action of these photocatalysts depends on their surface properties and morphology, i.e., on the properties of the semiconductor and noble metal and the interaction between them.

Titania with different morphologies such as NPs, nanotubes (TNT), nanowires, and nanospheres has been fabricated by various methods, e.g., hydrothermal reaction (HT), solvothermal, sol-gel, electrosynthesis and gas-phase methods [32], [52], [53], [54]. Octahedral anatase particles (OAPs), which expose eight equivalent and thermodynamically stable (1 0 1) facets, exhibit a very high level of photocatalytic activity, probably due to the preferential distribution of shallow electron traps (ETs) rather than deep ETs that results in a low rate of recombination of charge carriers [55], [56]. Although OAPs have a high level of photocatalytic activity, they are inactive under visible light (as is bare titania). Therefore, their surface modification with NPs of plasmonic metals, i.e., gold (Au), silver (Ag), platinum (Pt) and copper (Cu), has been investigated for decomposition of both chemical and microbiological pollutants under a broad range of irradiation conditions.

## 2 Experimental

### 2.1 Preparation of bare and metal-modified OAPs

Potassium titanate nanowires (TNWs; Earthclean Tohoku Co. Ltd), prepared by hydrothermal reaction (HT) of Evonik P25 titania (Nippon Aerosil) and potassium hydroxide solution (17 mol L^−1^) at 383 K for 20 h [57], were used for fabrication of titania samples containing OAPs. The TNWs (267 mg) were ultrasonically dispersed in Milli-Q water (40 mL) for 1 h, and then the obtained suspension was put into a 100-mL Teflon bottle into which an additional 40 mL of Milli-Q water was added. The bottle was sealed and placed in the outer sleeve of a stainless autoclave and then heated in an oven for 3–24 h (OAP sample heated for 6 h was used for further metal deposition.) at 433 K. The obtained suspension was dispersed by ultrasonication for 10 min and then centrifugally separated (12,000 rpm, 20 min). The white precipitates were collected and dried overnight under vacuum (353 K, 12 h).

For preparation of metal-modified OAPs, aqueous (MiliQ water) solutions of chloroplatinic acid (H_2_PtCl_6_·6H_2_O, 99.9%, Wako Pure Chemical Industries, Ltd.), chloroauric acid (HAuCl_4_·6H_2_O, 99.9%, Wako Pure Chemical Industries, Ltd.), copper sulfate (CuSO_4_·5H_2_O, 99.9%, Wako Pure Chemical Industries, Ltd.) and silver nitrate (AgNO_3_, 99.8%, Wako Pure Chemical Industries, Ltd.) were used as metal precursors. The codes of metal-modified OAP samples were defined as Pt/OAP, Au/OAP, Cu/OAP and Ag/OAP, respectively. Five hundred mg of an OAP product that had been heated for 6 h was used for each photodeposition, and the amount of each metal was calculated to be 0.5 wt% of titania. The weighed OAP powder was put into a Pyrex glass tube equipped with a magnetic stirrer, to which 25 mL of methanol (99.5%, Wako Pure Chemical Industries, Ltd.) aqueous solution (50 vol%, MiliQ water) was added. Then, the aqueous solution of metal salt was slowly dropped while being stirred. The suspension was gas-sparged (argon or oxygen) for 15 min. The tube was sealed with a rubber septum and photoirradiated with magnetic stirring (500 rpm) by a 400-W high-pressure mercury lamp (Eiko-sha) under thermostatic control at 298 K (details presented elsewhere [58]). The thus-obtained photocatalyst was centrifuged (15,000 rpm for 30 min), washed three times with methanol and three times with MiliQ water, and freeze-dried, and then the product was collected for further study.

Metal deposition on OAPs was carried out by two methods: under anaerobic conditions, i.e., proceeded by 15-min bubbling with argon (deaerated system) to remove oxygen from the tubes (Pt/OAP/Ar, Au/OAP/Ar, Cu/OAP/Ar and Ag/OAP/Ar), and under aerobic conditions, i.e., proceeded by 15-min bubbling with oxygen (aerated system) for efficient oxygen adsorption on OAPs (Pt/OAP/O_2_, Au/OAP/O_2_, Cu/OAP/O_2_ and Ag/OAP/O_2_). At the beginning of this photodeposition, photogenerated electrons are mainly consumed by oxygen, which hinders the reduction of metal cations and thus formation of metallic NPs.

### 2.2 Characterization of samples

The morphology was studied by scanning electron microscopy (SEM; JEOL JSM-7400F), scanning transmission electron microscopy (STEM, HITACHI HD-2000) and transmission electron microscopy (TEM, JEOL JEM-2100F). XRD analysis was performed using the Rigaku intelligent X-ray diffraction SmartLab system equipped with a sealed tube X-ray generator (a Cu target). Crystallite size of anatase was estimated from the corrected width of an anatase 1 0 1 diffraction peak using the Scherrer equation. Aspect ratio was calculated from the ratio of crystalline sizes estimated from the widths of diffraction peaks of (0 0 4) and (1 0 1). The oxidation states of elements and surface composition of samples were determined by XPS on a JEOL JPC-9010MC (MgKα X-ray) spectrometer. Photoabsorption properties of products were determined by diffuse reflectance spectroscopy (DRS) on a JASCO V-670 spectrophotometer equipped with a PIN-757 integrating sphere. Barium sulfate and bare OAP products were used as references.

### 2.3 Evaluation of photocatalytic activity

Photocatalytic activities were tested under UV/vis irradiation for (a) oxidative decomposition of acetic acid (99.7%, Wako Pure Chemical Industries, Ltd.) and (b) anaerobic dehydrogenation of methanol (99.5%, Wako Pure Chemical Industries, Ltd.). In each experiment, 50 mg of the photocatalyst was suspended in 5 mL of aqueous (MiliQ water) solution containing (a) 5 vol% acetic acid and (b) 50 vol% methanol and then photoirradiated under (a) air and (b) argon with magnetic stirring (1000 rpm). Photoirradiation was carried out in the same irradiation set-up as that used for metal photodeposition. Amounts of liberated CO_2_ (a) and H_2_ (b) in gas phase were determined by gas chromatography (TCD-GC). The visible light activity for oxidation of 2-proponol (99.7%, Wako Pure Chemical Industries, Ltd.) was evaluated by determination of the amount of acetone generated. Fifty mg of the photocatalyst was suspended in 5 mL of 2-propanol (5 vol%) and irradiated under magnetic stirring in a photoreactor equipped with a 300-W xenon lamp (CX-04E Inotech, Japan), quartz mirror, water filter and cut-off filter (Y45 (λ > 420 nm), Asahi Techno Glass). The photoreactor was described in detail in previous reports [49], [58]. The generated acetone was analyzed in liquid phase, after its filtration through a syringeless filter (Whatman Mini-UniPrep, PVDF), by gas chromatography (Shimadzu GC-14B, equipped with a flame ionization detector).

### 2.4 Evaluation of antibacterial property

Antimicrobial properties were investigated in the dark (control) and under irradiation with UV and/or visible light using xenon lamp with UV-35 (λ > 350 nm) and Y-45 (λ > 420 nm) cut-off filters, respectively. A suspension of 50 mg of the photocatalyst and 7 mL of microorganisms suspension in sterile 0.9% saline solution (NaCl) was used. The following microorganisms were used: *Escherichia coli* K12 bacteria from ATCC29425 and *Candida albicans* fungi isolated from patients with a poor immune system (throat smear). The density of bacteria was 0.5 in McFarland standards, which corresponds to ca. 1.5 × 10^8^ colony-forming units (CFU) mL^−1^, and 1 x 10^4^ cells mL^−1^ for fungi.

Antimicrobial tests were performed by the plate counting method as follows: 0.5 mL of the suspension was taken after 0.5, 1, 2 and 3 h of reaction. Serial dilutions were prepared and spread over agar media PCA (Plate Count Agar from Becton, Dickinson and Company) for *E. coli* and MEA (Malt Extract Agar from Merck Millipore Corporation) for *C. albicans*. The plates were incubated overnight at 310 K, and then the number of colonies was counted to determine the number of viable cells (as log N/N_0_).

## 3 Results and discussion

### 3.1 Preparation and characterization of bare OAPs

**Fig. 1:**
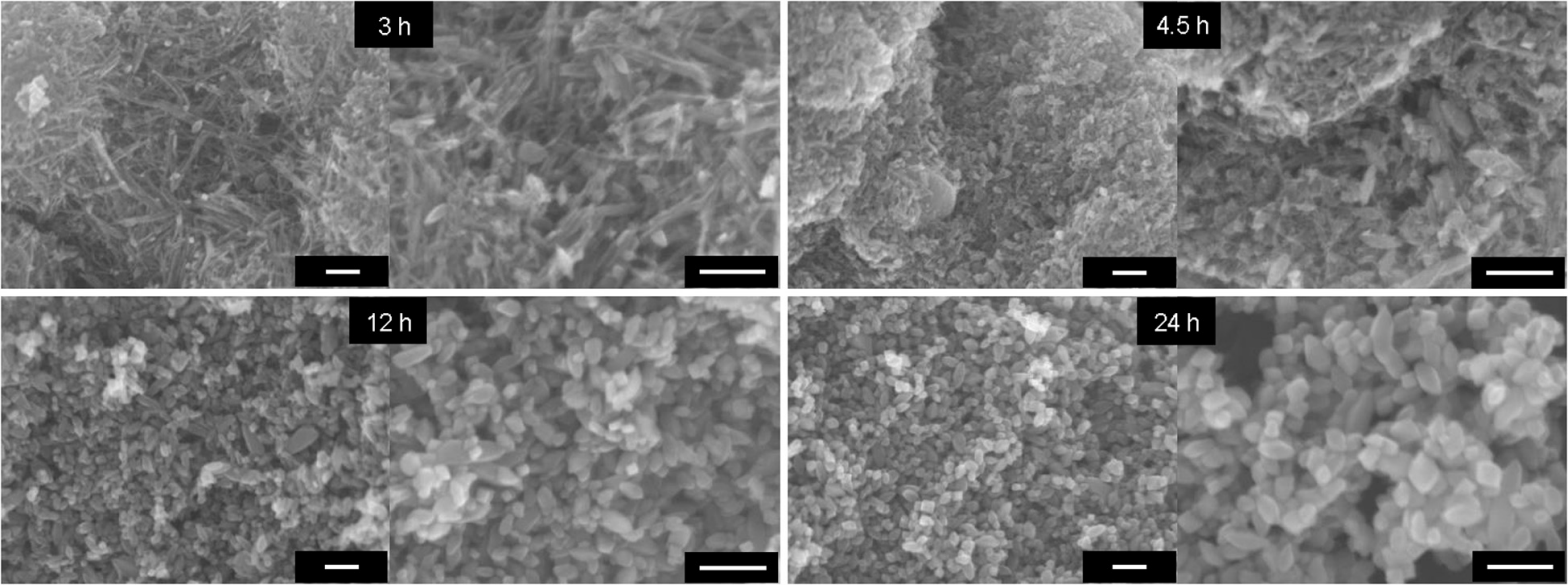
SEM images of samples prepared with various durations of HT process (3, 4.5, 12 and 24 h) with (left) low magnification and (right) high magnification. Scale bars correspond to 100 nm.

**Fig. 2:**
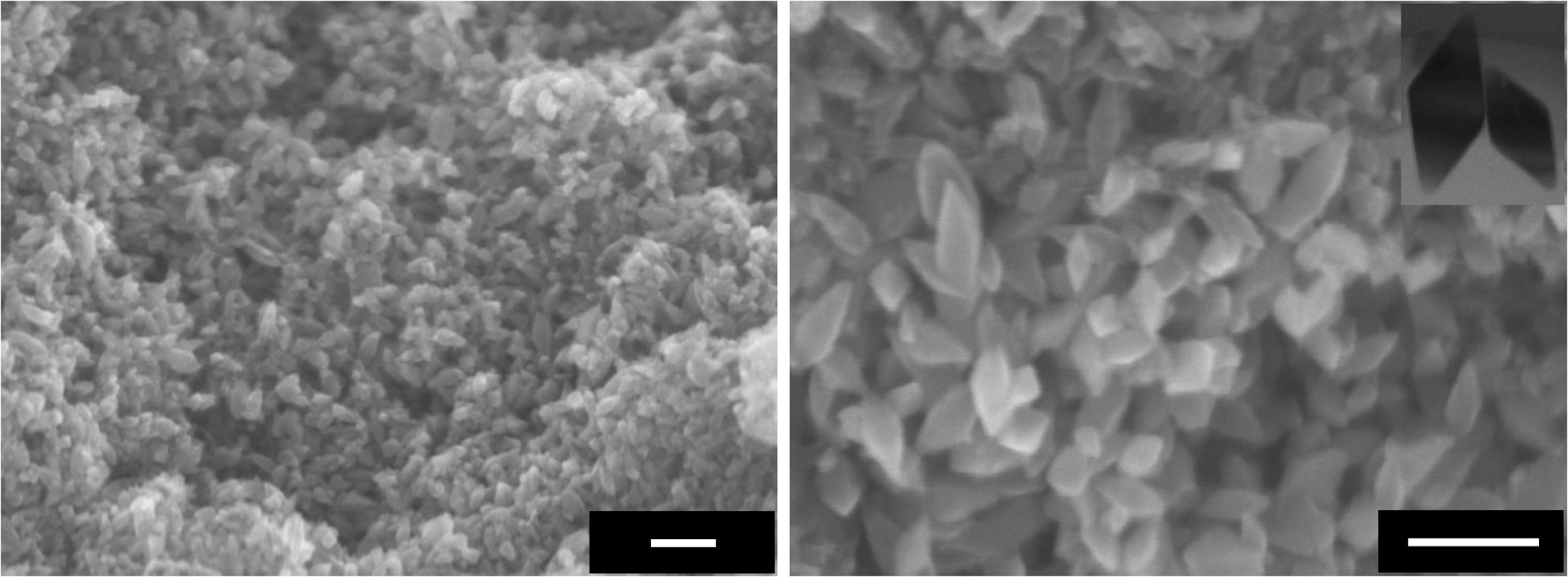
SEM images with (left) low magnification and (right) high magnification of the bare OAP-containing sample prepared with HT treatment for 6 h. Scale bars correspond to 100 nm. Inset shows TEM image of OAPs.

Preparation of titania samples containing OAPs from TNWs by the ultrasonication-hydrothermal method (US-HT) was described in detail previously [55], [56], [59]. It has been shown that duration and temperature of the HT process [56], pre- and post-treatment operations (US duration, calcination and grinding) [59], pH value of the reaction suspension and the content of water and TNWs [60] significantly influenced the properties of titania. For example, a higher rate of conversion of TNWs was obtained with either extension of the duration or increase in the temperature of the HT process, and the best morphology (highest content of facetted NPs in the final product) was obtained for optimal conditions of the US-HT process, e.g., 1-h US, 6-h HT at 433 K (depending on the properties of TNWs) [56]. In the present study, five durations of the HT process (3, 4.5, 6, 12 and 24 h) were tested for preparation of titania containing OAPs. It was found that durations of 3 h and 4.5 h were not sufficient for complete conversion of TNWs into OAPs, as is clearly shown in Fig. 1, where a nanowire structure of TNWs is the predominant morphology instead of an octahedron shape of anatase titania. With increase in duration of the HT process, an octahedron morphology became predominant (Fig. 1, Fig. 2). It is thought that the TNWs are dissolved and re-crystallized into anatase during the HT process and that the HT system finally reaches a dynamic equilibrium between precipitation and dissolution of TNWs, resulting in some wire-like structures remaining in the final product. The conditions of the HT process influence this dynamic equilibrium. The sample prepared with HT treatment for 6 h (shown as an enlargement in Fig. 2) was used for further modification with metals to keep the duration of the HT process as short as possible for possible future application.

**Fig. 3:**
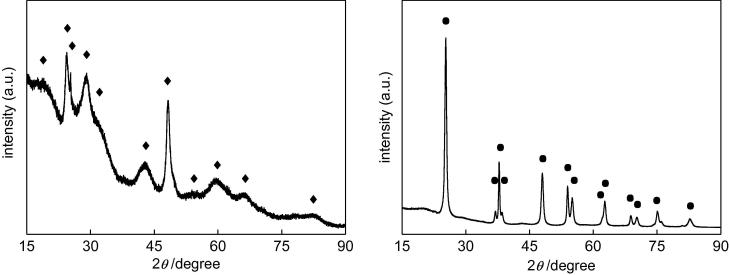
XRD patterns of (left) TNWs and (right) OAPs: ♦ – K_2_Ti_8_O_17_, ● – anatase titania.

Crystallographic data confirmed that the observed octahedron shape was caused by the presence of anatase as shown in Fig. 3 (right). Peaks in the XRD pattern of the precursor (TNWs) were less intense and broader, indicating low crystallinity of K_2_Ti_8_O_17_ (Fig. 3, left). As was observed in a previous study [56], growth of crystals was observed with prolongation of the HT process. The crystal size of anatase (1 0 1) increased from 9.6 to 24.4 nm with an increase in the duration of the HT process from 3 to 24 h (detailed data are shown in Table S1).

The observed absorption edge of all bare OAPs was typical for non-modified anatase samples, i.e., at ca. 400 nm, as shown in the Supplementary File (Fig. S1).

### 3.2 Modification of OAPs with noble metals

**Fig. 4:**
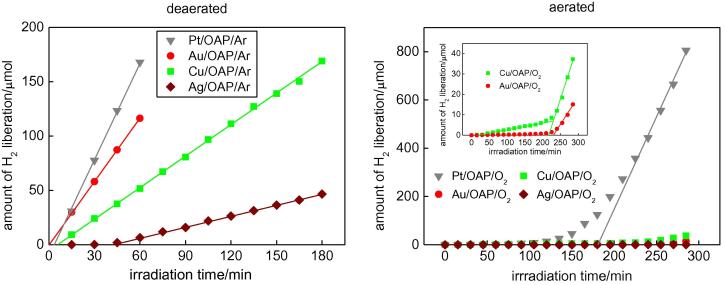
Methanol dehydrogenation during metal photodeposition on OAPs under (left) anaerobic and (right) aerobic conditions.

The photodeposition method was used for modification of OAPs with metallic nanoparticles (NPs). Usually, photodeposition is performed under anaerobic conditions to avoid electron scavenging by oxygen and thus to use all of the photo-excited electrons for reduction of metal cations. In addition, methanol (or another hole scavenger) is added to the titania suspension to hinder the recombination of electrons with holes. However, we have recently found that photodeposition of platinum in the initial presence of oxygen results in the formation of finer Pt NPs that are more uniformly dispersed on the titania support than that performed under anaerobic conditions [60]. In the present study, both kinds of photodeposition were used, i.e., under anaerobic conditions (15-min Ar pre-bubbling) and under aerobic conditions (15-min O_2_ pre-bubbling), to investigate their influence on the properties and photocatalytic activities of metal-modified OAPs. As expected, the presence of oxygen significantly hindered the reduction of metal cations and deposition of metallic NPs on OAPs, as shown in Fig. 4.

The induction period (intersection with the x-axis), during which NPs of noble metals are formed, depends on the properties of metal and titania (e.g., amount, kind and distribution of electron traps). In the case of platinum and gold, deposition under anaerobic conditions is very fast, and only a few minutes is sufficient for the reduction of all metal cations and formation of NPs. This is evident by linear evolution of hydrogen at a constant rate [49], [61], [62]. The complete deposition of Au by the photodeposition method has already been confirmed for a wide range of gold contents from 0.05 to 10 wt% by atomic absorption spectroscopy [61]. Similarly, in the present study, photodeposition of Au and Pt was very fast and less than 5 min was sufficient for their deposition on OAPs. On the other hand, deposition of Cu and Ag needed longer times: ca. 8 min and 45 min, respectively. The rate of methanol dehydrogenation correlated with reduction facility of metal cations (induction period), i.e., the shorter the induction period was, the higher was the rate of hydrogen generation. It is thought that rates of formation of metallic deposits and methanol dehydrogenation correlate with the difference between the work function of metal (Ag = 4.26 eV, Cu = 4.7 eV, Au = 5.1 eV, Pt = 6.35 eV) and the electron affinity of titania (4.0 eV) [75], i.e., the electronic potential barrier (Schottky barrier) generated by the band alignment at the metal-semiconductor heterojunction. More detailed discussion of photocatalytic activity is presented in Section 3.4.

Under aerobic conditions, much longer induction periods were needed for metal photodeposition and for linear evolution of hydrogen, i.e., ca. 180, 215, 230 and >300 min for Pt, Cu, Au and Ag, respectively. At first, excited electrons were mainly captured by oxygen, and only a slight reduction of metal cations was observed, as shown in the right part of Fig. 4. Oxygen consumption (shown in Fig. S2) correlates with respective induction periods, i.e., the fastest oxygen consumption (ca. 120 min) was achieved during Pt deposition. Interestingly, a different tendency was observed for the two kinds of photodeposition of Cu and Au, i.e., a shorter induction period for Cu than that for Au (inset of Fig. 4, right) under aerobic conditions, suggesting that instead of Cu, copper oxide (CuO, 5.3-eV work function) was firstly deposited on OAPs. Interestingly, in the case of Cu and Ag deposition, two rates of hydrogen evolution were observed: (1) with a short induction period (“initial” induction period of ca. 40–50 min) linear evolution of hydrogen was observed at a very low rate (ca. 0.04 and 0.002 μmol min^−1^ for Cu and Ag, respectively) and then (2) a significant increase in the reaction rate was observed after complete oxygen consumption (change from aerobic to anaerobic conditions). Therefore, it is thought that oxides of Ag and Cu are formed under aerobic conditions and then they are reduced to zero-valent metal under anaerobic conditions.

### 3.3 Characterization of metal-modified OAPs

**Fig. 5:**
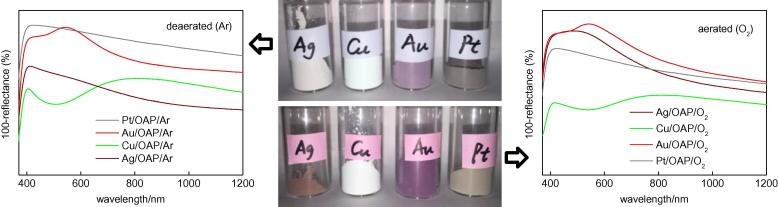
Photoabsorption properties of modified OAPs samples prepared under (left) anaerobic and (right) aerobic conditions with bare OAPs used as a reference.

Photoabsorption properties (DRS) and photographs of all modified samples are shown in Fig. 5. Since the LSPR position of Pt and Ag is near the band edge of titania, DRS spectra were taken with bare OAPs as a reference (to eliminate titania absorption properties). DRS spectra for barium sulfide as a reference were also taken and are shown in the Supplementary File (Fig. S3). It should be pointed out that the position of LSPR of noble metals depends on the kind of noble metal, the properties of NPs such as their size and shape, and the environment (diffractive index). In general, LSPR at shorter wavelengths is observed for spherical and small NPs, and change in the shape of NPs or increase in the size of NPs results in a bathochromic shift of LSPR. For example, LSPR of spherical NPs of Pt, Ag, Au and Cu appears at ca. 400 nm [63], 430 nm [64], 540 nm [65] and 560 nm [66], respectively.

Pt and Au are chemically stable and their exposure to an ambient environment after preparation does not change their surface properties. On the other hand, Ag and Cu are easily oxidized, and their oxides are usually observed on the surface of NPs, regardless of the preparation method [62], [67]. For example, titania modification by strong radiolytic reduction of Cu^2+^ resulted in the formation of CuO/TiO_2_ [68], and even oxidation of zero-valent Cu by oxygen diffused into water was reported [66]. In this regard, it was expected that both metallic and oxidized forms of Ag and Cu could be detected during their characterization.

In our study, contrary results were obtained for particular metals by changing the photodeposition method from anaerobic to aerobic conditions, i.e., an increase in photoabsorption intensity (DRS and color darkening, Fig. S4) was observed for Au and Ag samples, and a slight decrease was observed for Pt and Cu samples. The shape of DRS spectra was almost unchanged for Au and Pt, slightly changed for Cu, and significantly changed for Ag (Fig. S4). The broadening of LSPR of silver indicated that aerobic conditions resulted in the formation of more polydispersed silver NPs. It should be noted that DRS spectra consisted of both absorption and scattering. Therefore, less intensive DRS spectra of Pt/OAPs at longer wavelengths confirmed our previous findings that smaller Pt NPs were formed under aerobic conditions [60]. In the case of Au, there was almost no change in LSPR. The intensity was stronger, but there was less scattering at longer wavelengths (clearly shown in Fig. S4), suggesting the formation of a smaller amount of larger NPs. In contrast, no LSPR peak was observed for Cu/TiO_2_, confirming its easy oxidation in an ambient environment (after preparation) resulting in the characteristic absorption band of CuO near IR [68], [69], [70]. The slightly green color of Cu/OAP/Ar suggests that Cu can exist also in the form of [Cu(OH)]_2_CO_3_ (or less possible CuO_2_, due to its instability). It should be pointed out that just after photodeposition (under aerobic and anaerobic conditions), the color of Cu samples was violet (Fig. S5), confirming that metallic copper was formed on titania. Interestingly, in the case of deposition under aerobic conditions, black deposits of CuO were first observed (Fig. S5, right) and then zero-valent copper (violet color) was formed after oxygen consumption.

XRD was performed to determine crystalline composition and crystal sizes of metallic deposits, and the data obtained are summarized in Table 1. Since a small amount of metals was used for modification (0.5 wt%) and their sizes were very small, no clear XRD peaks for metallic deposits were observed, with the exception of Au/OAP/O_2_, which showed characteristic gold features (Fig. S6). An Ag peak was not clearly observed even for a much larger amount of deposited metal (2 wt%) when small anatase NPs (of sizes to those of OAPs) were used as a support [62]. Therefore, Rietveld refinement was used for detailed data analysis. In the case of Pt and Au, zero-valent deposits were clearly identified, while less noble metals (Ag and Cu) existed mainly in the form of their oxides (Ag_2_O and CuO), as was suggested from photodeposition data (Fig. 4, right). The method used for preparation influenced the sizes of metallic deposits, i.e., slightly smaller sizes of Pt and much larger sizes of other metals were obtained after changing the deposition conditions from anaerobic to aerobic conditions. An increase in crystal size is surprising since slowing down the deposition process by the presence of oxygen should result in the formation of smaller metallic deposits, as has been observed for Au deposition on various commercial titania samples (unpublished data). Some possible reasons for the estimated larger crystal sizes of metallic deposits formed under aerobic conditions (except Pt) are as follows. (1) The growth of crystallites is predominant instead of the formation of new crystallites (and their dispersion on OAPs), due to special features of OAPs (i.e., octahedron morphology, high crystallinity, small amount of deep electron traps and small crystal size). A recent study has shown that metallic NPs could preferentially be deposited on facets other than (1 0 1), i.e., on imperfect anatase crystals [60]. (2) Polydispersity in the size of metallic deposits results in overestimation of crystallite sizes, since small gold nano-clusters (nanometer-sized) are undetectable by XRD (Determination of size distribution by detailed microscopic observation should be performed for reliable evaluation.). (3) In the case of less noble metals (Ag and Cu) deposited under aerobic conditions, respective oxides are formed first, but there are no metallic deposits, which are formed under anaerobic conditions. Therefore, it is possible that anaerobic conditions result in the formation of small zero-valent deposits, which are surface-oxidized by the contact with the ambient atmosphere. On the other hand, under aerobic conditions, much larger metallic oxides (and also zero-valent metals) are directly formed under irradiation.

STEM was used to investigate the morphology of modified samples, and exemplary images for Au/OAP/Ar and Ag/OAP/Ar samples are shown in Fig. S7. It is clear that mainly small Ag and Au NPs of ca. 2–10 nm in size were deposited on titania. Generally, Au formed slightly larger and aggregated NPs than did Ag, as is shown in Fig. S8 (5–10 nm and 2–5 nm, respectively). Formation of larger Au NPs than Ag NPs by the photodeposition method has been reported for other titania samples [62], [67].

To characterize the surface compositions of samples, oxygen, titanium, carbon and noble metals (Au, Ag, Pt and Cu) were analyzed by XPS and the data obtained are summarized in Table S2. It was found that the ratio of oxygen to titanium exceeded that of the chemical formula of titania (O:Ti = 2), reaching ca. 2.1–2.5 and ca. 5 for samples prepared with shorter (3–6 h) and longer (12 and 24 h) HT processes, respectively, which is reasonable since extension of the duration of the HT process should result in enrichment of the titania surface with hydroxyl groups. Various studies have shown an excess of oxygen on the titania surface, e.g., a ratio of 4.6 for titania samples prepared by the microemulsion method [71] and a ratio of 2.5 for titania prepared by laser ablation [72].

**Fig. 6:**
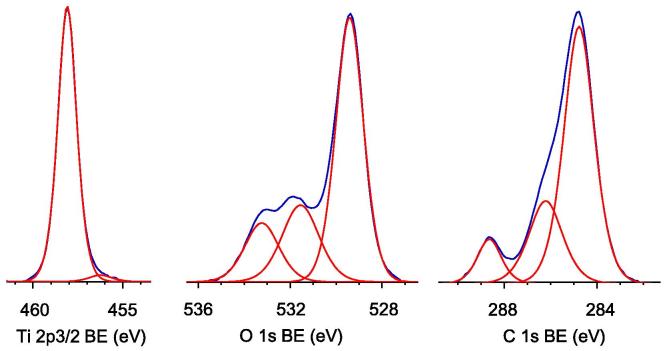
XPS results for Ti 2p_3/2_, O 1s, C 1s of bare 6 h OAP.

Oxidation states of all elements were analyzed by deconvolution of XPS peaks. Exemplary data for bare OAPs sample prepared with HT treatment for 6 h are shown in Fig. 6. Titanium in all bare OAPs samples existed mainly in Ti^4+^ form (95.9–99.4%), and extension of the duration of HT treatment resulted in the formation of a more crystalline TiO_2_ structure with a smaller amount of oxygen vacancies, i.e., Ti^3+^ (detailed data shown in Table S2). Similarly, the form of carbon did not significantly differ in titania samples. Carbon 1 s region was deconvoluted for three peaks at binding energies (BE) of ca. 284.8 eV, 286.1 eV and 288.6 eV, which could be assigned to C-C, C-OH and C=O states, respectively, and obtained contents were 65.3–73.1%, 19.0–24.4% and 5.7–10.3%, respectively. Carbon originates from the atmosphere during preparation of samples for XPS measurements and is always detected in titania samples [73]. In contrast to titanium and carbon, the form of oxygen differed significantly between samples, as shown in Fig. S9. According to literature reports, O 1s peak could be composed of three to five different oxygen species such as Ti-O bonds in TiO_2_ and Ti_2_O_3_, H-O bonds in hydroxyl groups, carbon bonds (C-O and C=O) and adsorbed water [74]. In our study, three peaks were observed after deconvolution of O 1s peak in all samples at BE of ca. 529.4 eV, 531.6 eV and 533.2 eV. The first peak is related to oxygen in the crystal lattice of TiO_2_, the second peak is related to C=O, Ti_2_O_3_, and/or OH groups bound with two titanium atoms, and the third peak is related mainly to hydroxyl groups bound to titanium and carbon (Ti-OH, C-OH) [75]. It is clear that an increase in the duration of the HT process resulted in enrichment of the titania surface with hydroxyl groups. This observation contradicts the results of a previous study performed for different commercial titania samples prepared by various methods (no HT) showing that a smaller specific surface area correlates with lower content of hydroxyl groups on the titania surface [73]. In our study, an increase in the duration of the HT reaction resulted in an increase in crystallite size (as shown in Table S1) and thus a simultaneous decrease in specific surface area [56]. Therefore, it is thought that the HT process is unique by allowing efficient contact between titania and water, which results in preparation of titania samples possessing an excess of hydroxyl groups on the surface.

**Fig. 7:**
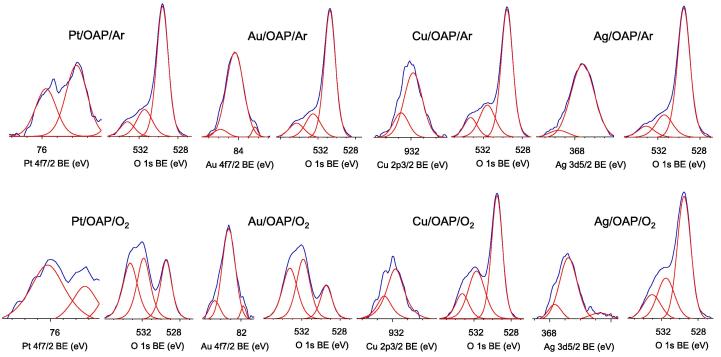
XPS results for Pt 4f_7/2_, Au 4f_7/2_, Cu 2p_3/2_, Ag 3d_5/2_ and O 1s for samples prepared under (top) anaerobic and (bottom) aerobic conditions.

The presence of metals was confirmed in all modified samples, as shown in Table S2 and Fig. 7. The content of metals exceeded the amount used for deposition (0.5 wt%) reaching ca. 3.5, 2.5, 2.2 and 3.1 wt% for Pt, Au, Cu and Ag, respectively, for samples prepared under anaerobic conditions. Enrichment of the titania surface with metals was expected and was previously reported [67], since metals were deposited on the titania surface, and the surface compositions of samples were determined by XPS analysis. Interestingly, the content of metals changed when photodeposition was performed under aerobic conditions. Larger amounts of Pt and Au (4.2 wt% and 4.7 wt%, respectively) and smaller amounts of Cu and Ag (1.3 wt% and 1.4 wt%, respectively) were obtained. This confirms previous data (DRS, photodeposition and partly XRD) suggesting that Pt and Au formed smaller NPs under aerobic conditions (greater covering of the titania surface, higher XPS peaks of metals) but that an oxide layer was formed (lower XPS peaks of metals) on the surface of less noble metals (Ag and Cu). This hypothesis is also confirmed by XPS data of the oxygen peak, as shown in Fig. 7. The form of oxygen did not significantly differ between samples prepared under anaerobic conditions (top line), but Au and Pt-modified OAPs prepared under aerobic conditions had a very large amount of hydroxyl groups on the titania surface (bottom line), similar to OAP samples prepared with an extended duration of the HT process (Fig. S9). In contrast, hydroxyl groups were not additionally formed on OAPs modified with Ag and Cu, since oxygen formed metallic oxides on the surface of these less noble metals. Deconvolution of Pt [76], [77], [78], Au [71], Cu [68], [79] and Ag [67], [68], [71] peaks confirmed that metal deposition under anaerobic conditions resulted in the formation of mainly zero-charged Au and Pt (ca. 90% and 61%, respectively) deposits and oxidized forms of Ag and Cu (Table 2). Changing the method for preparation to aerobic conditions resulted in the formation of NPs with more positive charges, even for Pt and Au. Therefore, it is proposed that hydroxyl groups could be adsorbed on both the titania surface and metallic deposits. The method of deposition had almost no influence on the oxidation state of Cu, and only slightly more Cu^2+^ than Cu^+^ was formed under aerobic conditions (23.9% under anaerobic conditions and 28.4% under aerobic conditions). Interestingly, preparation of Ag/OAP under aerobic conditions resulted also in the formation of Ag^2+^ (besides predominant Ag^+^) and a larger content of zero-charged Ag(0), which explains the stable brown color of this sample even after drying.

### 3.4 Photocatalytic activity

**Fig. 8:**
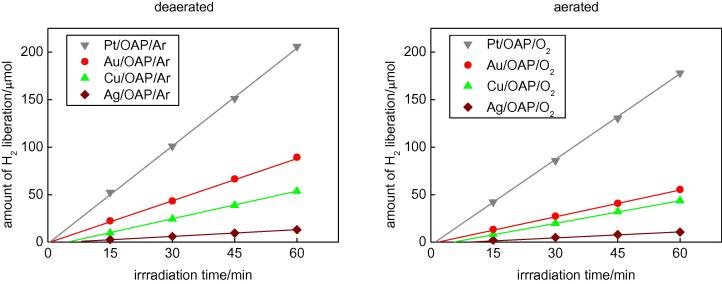
Anaerobic UV/vis dehydrogenation of methanol on OAPs modified with metals under (left) anaerobic and (right) aerobic conditions.

Photocatalytic activities of bare and modified OAPs were tested for two reaction systems under UV/vis irradiation (anaerobic dehydrogenation of methanol and oxidative decomposition of acetic acid) and for oxidation of 2-propanol under visible light irradiation (λ > 420 nm). Photoactivity tests for methanol dehydrogenation were carried out similarly to those for photodeposition of metals under anaerobic conditions but in smaller test tubes [49], i.e., at first oxygen was removed from the system by argon bubbling. Since metallic deposits were already present on the titania surface, almost no induction period was observed for Pt and Au and shorter induction periods were observed for Ag and Cu than during their deposition (Fig. 4), as shown in Fig. 8. It should be pointed that induction periods for Pt, Au and Ag were longer for samples prepared under aerobic conditions (0.9, 1.9, 9.4 min, respectively) than for samples prepared under anaerobic conditions (0.4, 0.7 and 7.5 min, respectively), suggesting that the positive charge on metallic deposits was first neutralized and then protons were reduced on the surface of zero-charged metallic deposits. Interestingly, the same induction periods were observed for two Cu-modified samples (ca. 5.6 min), which is not surprising since the surface properties of Cu/OAPs/Ar and Cu/OAPs/O_2_ photocatalysts were almost the same (Table 2), with Cu existing mainly in the form of its oxides.

A comparison of hydrogen evolution on two kinds of photocatalysts and comparison of enhancement of photocatalytic activity after OAP modification with metals are shown in Fig. S10. It was confirmed that bare titania was inactive for hydrogen evolution and that the presence of a metallic co-catalyst was necessary. It is known that the rate of hydrogen evolution depends on the kind of metallic co-catalyst and also on the structural properties of titania. For example, it was reported that the highest activities were observed for large rutile and fine anatase samples after their modification with gold [7], [18]. Considering metallic deposits, Pt is the most active for hydrogen evolution, followed by Au, Cu and Ag. Similar results have already been reported for Au, Ag and Pt-modified titania [37], [80] and for Au, Cu and Ag-modified titania [81]. It has been proposed that the lowest activity of silver-modified titania originated from its smallest work function, since the greater the difference between the metal work function and the electron affinity of titania is, the higher is the electronic potential barrier (Schottky barrier) generated by band alignment at the metal-semiconductor heterojunction. The higher the Schottky barrier is, the higher is the transfer and trapping of photogenerated electrons by metal [82], and thus the higher is the hydrogen generation rate. Therefore, the work functions of Ag, Cu, Au, Pt and titania of 4.26, 4.7, 5.1, 6.35 eV and 4.0 eV [83], respectively, correlated with their efficiency for hydrogen generation. Activation overpotential for hydrogen evolution on the surface of a co-catalyst should also be considered. Indeed, the values of overpotential correlate with resultant activities, with the smallest value of −0.05 V for Pt, the next smallest of −0.24 V for Au, and the largest values for less active metals, i.e., −0.48 V for Ag and Cu. Since Cu is mainly present in an oxide form, the overpotential for hydrogen evolution on Cu_2_O and CuO should be considered. The co-catalytic function of CuO (electron scavenging, similar to zero-valent noble metals) has already been reported for CuO/WO_3_ photocatalysts, where a multiple-electron reaction on CuO was proposed [84]. The participation of Cu oxides (CuO and Cu_2_O) in hydrogen evolution should also be considered since activity of these semiconductors has already been reported for ethanol dehydrogenation [85].

Interestingly, all of the rates of hydrogen evolution for photocatalysts prepared under aerobic conditions are slightly lower than those for photocatalysts prepared under anaerobic conditions (Figs. 8 and S10). It is thought that the presence of adsorbed oxygen/hydroxyl groups (Fig. 7, Table S2) on both titania and metallic deposits hindered methanol and proton adsorption and thus their reactions with photogenerated holes and electrons, respectively.

**Fig. 9:**
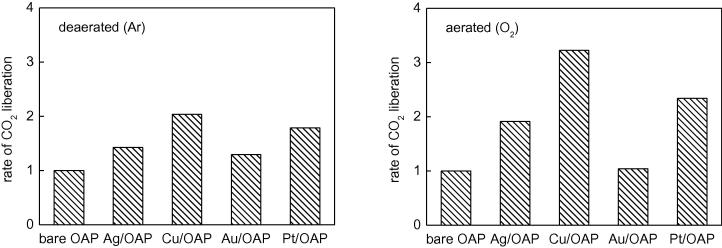
Aerobic UV/vis decomposition of acetic acid on OAPs modified with metals under (left) anaerobic and (right) aerobic conditions.

The photocatalytic activities of bare and metal-modified OAPs in another reaction system, oxidative decomposition of acetic acid, are shown in Fig. 9 (as relative rates in comparison to bare OAP) and Fig. S11 (The photocatalytic activities of bare OAPs prepared with different durations of the HT process will be discussed further with antibacterial properties, Fig. 11.). It is clear that modification of OAPs with noble metals resulted in significant enhancement of photocatalytic activity. Interestingly, enhancement in activity did not correlate with work function of metals, suggesting that not only the formation of a Schottky barrier but also other properties were crucial. Previous studies showed that the content of surface hydroxyl groups influenced the photocatalytic activity, as a source of reactive oxygen species (ROS) [86], [87]. Therefore, the highest photocatalytic activity of Cu/OAP correlated well with the largest amount of hydroxyl groups on its surface. It must be pointed that in contrasat to methanol dehydrogenation, decomposition of acetic acid was performed under aerobic conditions, and thus it is highly probable that less noble metals (Ag and Cu) existed mainly in their oxide forms. Therefore, it is proposed that the heterojunction between two/three oxides, i.e., titania and CuO/Cu_2_O and titania and Ag_2_O/AgO, could result in inhibition of the recombination of charge carriers, as has already been reported for such coupled semiconductors [68], [69], [88], [89].

Interestingly, samples prepared under aerobic conditions had much higher photocatalytic activity than those prepared under anaerobic conditions, and the correlation between enhancements of photocatalytic activities after metal deposition by the two preparation methods was linear (Fig. S12). It is proposed that surface enrichment with oxygen and hydroxyl groups (Table S2), and thus more efficient generation of ROS, as has recently been proposed for titania modified with rare earth elements [86], is responsible for the high level of photocatalytic activity of samples prepared under aerobic conditions.

**Fig. 10:**
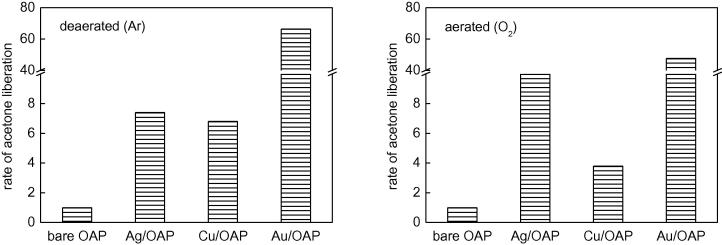
Aerobic vis oxidation of 2-propanol on OAPs modified with metals under (left) anaerobic and (right) aerobic conditions.

All of the modified photocatalysts showed activity under visible light irradiation and inactivity in the dark with the exception of Pt/OAP. Pt/OAP showed an extremely high level of activity (ca. 2 orders higher than that of Au/OAP), mainly due to its “dark” catalytic activity (>90% activity). The dark activity of Pt/OAP is a very interesting matter for further detailed study, but this issue is not included in this paper (to avoid complex and confusing discussion). Therefore, only photocatalytic activities of bare OAPs and OAPs modified with Au, Cu and Ag are presented in Fig. 10. Visible activity of titania modified with Au and Ag (due to activation of their plasmon resonance (LSPR)) is well known and is now being extensively studied. It was proposed that light absorption at broader wavelengths (wide LSPR) resulted in enhanced photocatalytic activity since more photons (of different energies) could be absorbed and then efficiently participate in overall photocatalytic activity [49]. Therefore, change in the deposition conditions (from anaerobic to aerobic conditions) that resulted in enhanced photocatalytic activity of Ag/OAP and lower activity of Au/OAP correlated well with photoabsorption properties, i.e., broader LSPR for Ag/TiO_2_ and narrower for Au/TiO_2_. Much lower photocatalytic activity of Ag/OAP than that of Au/OAP might have been caused by the experimental conditions, i.e., irradiation with wavelengths longer than 420 nm (to ensure inactivity of bare titania) and thus cutting of a large part of the LSPR of Ag.

The visible activity of Cu/OAP should result from excitation of Cu oxides possessing much narrower band-gap than that of titania (2.0–2.2 eV for Cu_2_O and 1.3–1.6 eV of CuO [90]), due to the lack of characteristics of zero-valent Cu (LSPR at ca. 560 nm and Cu(0) by XPS). It was also proposed that electrons in the valence band of titania could be excited to Cu^II^ species through an interfacial charge transfer (IFCT) process [91]. Photocatalytic activity under visible light irradiation of single Cu oxides (CuO and Cu_2_O) [85], hybrid CuO-Cu_2_O arrays [90], and Cu oxides coupled with other semiconductors (Cu_x_O/TiO_2_ [91], [92], CuO/TiO_2_ [68]) has already been reported for hydrogen evolution and oxidation of organic compounds. The lower photocatalytic activity of Cu/OAP/O_2_ than that of Cu/OAP/Ar could result from slightly different contents of Cu_2_O and CuO in these samples, which could influence the transfer of excited electrons from Cu_2_O to CuO/TiO_2_ [69], [90]. It is also likely that the different composition of the Cu/OAP/Ar sample (green color, due to [Cu(OH)]_2_CO_3_ or CuO_2_ incorporated in the surface layer of OAP) is responsible for the enhanced visible activity. Further research on this matter is currently being conducted.

### 3.5 Antimicrobial activity

**Fig. 11:**
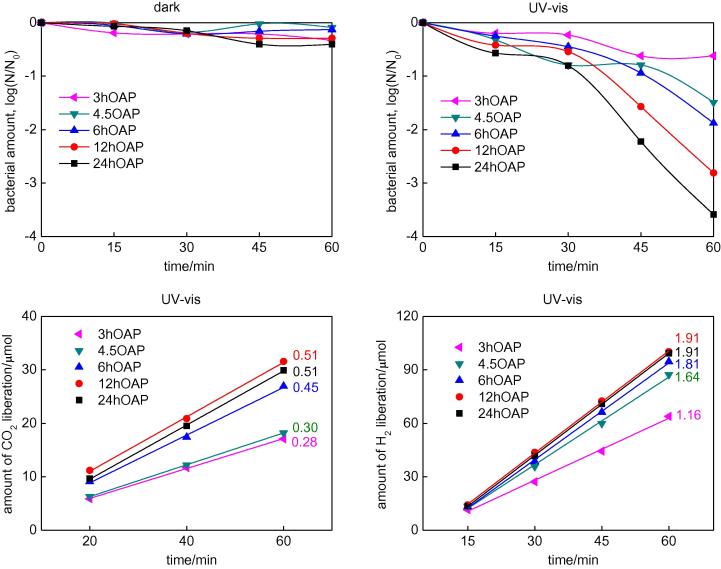
Activity of bare OAPs prepared with various durations of the HT process for: (top) *E. coli* (left) in the dark and (right) under UV/vis irradiation (Y35 filter), (bottom, left) acetic acid under UV/vis irradiation and (bottom, right) methanol under UV/vis irradiation (2 wt% of Pt *in-situ* deposited).

**Fig. 12:**
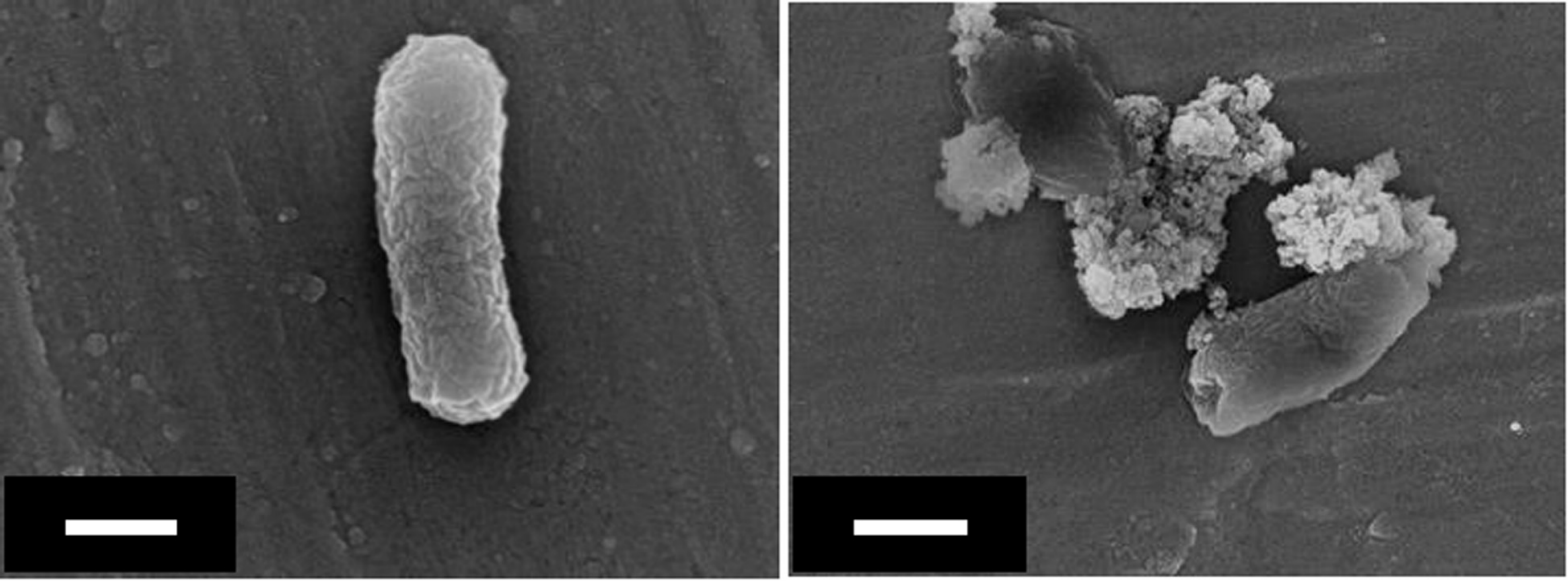
SEM images of *E. coli* (left) before and (right) after 1 h of UV/vis irradiation in the presence of 6 h OAP. Scale bars: 500 nm.

At first, antibacterial (against *E. coli*) properties of bare OAPs were tested in the dark and under UV/vis irradiation (λ > 350 nm), and the data obtained are shown in Fig. 11 (top). A comparison of the activities of those samples for acetic acid decomposition and dehydrogenation of methanol (in the presence of *in-situ* deposited Pt) under UV/vis irradiation is also shown in the bottom part of Fig. 11. It is clear that bare OAPs do not have significant activity in the dark, while anatase is activated by UV irradiation and the ROS that were formed are responsible for bacteria inactivation, as has been reported for other titania samples [93]. SEM images of healthy and destroyed bacterial cells before and after 1-h irradiation in the presence of OAPs prepared with HT treatment for 6 h are shown in Fig. 12.

Under UV/vis irradiation the antibacterial activity of titania is directly correlated with its structural properties, which was confirmed by a similar tendency in two other systems for decomposition of chemical compounds (Fig. 11, bottom). The lowest activities in all tested reaction systems were observed for OAP samples prepared with shortest duration of HT treatment, which is not surprising since these samples contained a larger content of unreacted TNWs (Fig. 1). With increase in duration of HT treatment, efficiency of conversion of TNWs into anatase increased, resulting the highest activity of samples prepared for 24 h. It should be pointed that optimal conditions of the HT process that resulted in high crystallinity and sufficient surface area of samples were obtained for various temperatures and durations of the HT process depending on the precursor properties. Our previous study performed for different TNWs showed that HT treatment for 6 h resulted in the best morphology and resultant activities, while extension of the duration of HT treatment resulted in aggregation of OAPs [55]. At present, it is clear that the best properties of OAPs are obtained for samples prepared with HT treatment for 12 h and 24 h (same activities for methanol dehydrogenation and acetic acid decomposition). However, antibacterial properties of the samples differed and the best activity was obtained for samples prepared with a longer duration of the HT process. The crystallite sizes of the samples did not differ significantly, but the aspect ratio of the 24 h OAP sample was smaller than that of the 12 h OAP sample (Table S1). Therefore, the presence of an additional (0 0 1) facet might be beneficial for bacteria inactivation, which is reasonable due to the different distributions of charges on the (1 0 1) and (0 0 1) facets (unpublished data). This hypothesis, i.e., the influence of facet content on antibacterial properties, will be investigated in detail in our future study.

**Fig. 13:**
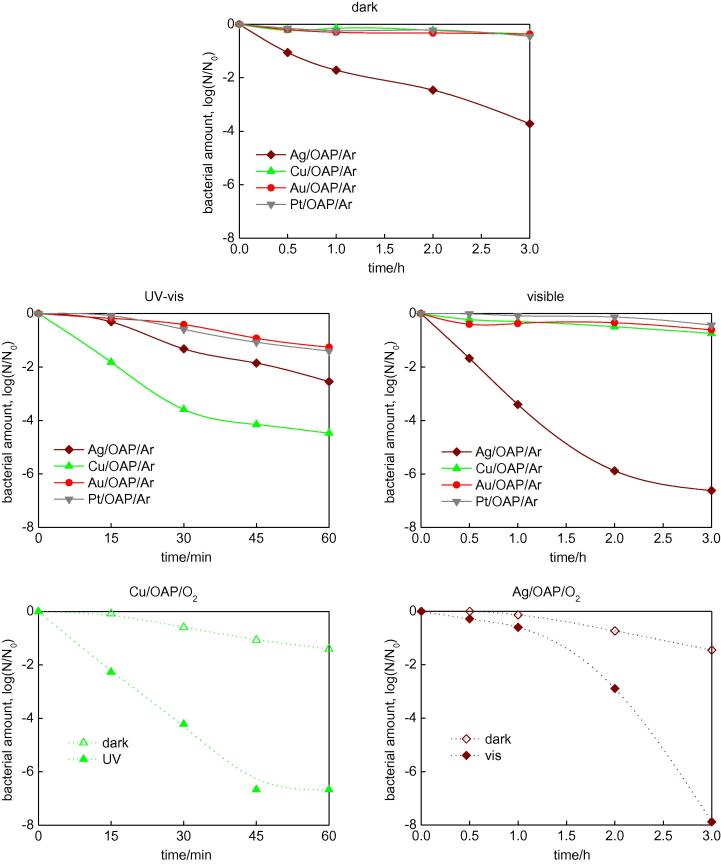
Antibacterial activity of OAPs modified with metals: (top-middle) under anaerobic conditions: (top) in the dark, (middle, left) under UV/vis (λ > 350 nm), (middle, right) under vis (λ > 420 nm); (bottom) under aerobic conditions: (left) Cu/OAPs/O_2_ in the dark and under UV/vis (λ > 350 nm), (right) Ag/OAPs/O_2_ in the dark and under vis (λ > 420 nm).

The antibacterial activities of OAP samples modified with noble metals (modification under anaerobic conditions) in the dark and under irradiation with UV and/or vis are shown in Fig. 13 (top). In the dark, only Ag/OAP showed antibacterial properties, which is not surprising considering the excellent antimicrobial properties of Ag that have been known since ancient times [94], [95]. Various studies have been performed to determine the mechanism underlying the action of silver. It was proposed that especially in the case of Gram-negative bacteria (e.g., *E. coli*), Ag NPs could adsorb and accumulate on the outer membrane, creating gaps in the integrity of the bilayer, which results in an increase in permeability and finally in death of bacteria cells [96]. It has been proposed that smaller size of NPs and positive zeta potential promote interactions that allow penetration of NPs in bacteria membranes. In addition, the release of Ag cations from Ag NPs in water or during penetration of NPs into cells has been suggested [97]. Although NPs have higher antibacterial activity than that of the free ions of Ag, the overall antibacterial properties are attributed to both the physical properties of NPs and the elution of Ag cations [95].

Under UV/vis irradiation, only OAPs modified with Cu showed significant enhancement of antibacterial properties. Antibacterial properties of Cu-modified titania has recently been reported, and different mechanisms have been proposed. For example, damage of the outer cell membrane by ROS generated on irradiated titania with simultaneous infiltration of Cu ions across the cell membrane was reported [98], [99]. In contrast, Qiu et al. reported inactivity of free Cu ions (Cu^+^ and Cu^2+^) and claimed that only titania modified with solid-state Cu_x_O nanoclusters (possessing Cu^+^) showed enhanced activity in the dark and under irradiation with visible light [91]. The ratio of Cu^+^ to Cu^2+^ in CuxO nanoclusters of 1.3 was proposed as an optimal ratio for the best antibacterial properties. Therefore, the influence of Cu properties on antibacterial properties of Cu/OAP/O_2_ was also investigated, and data are shown in Fig. 13 (bottom, left). Interestingly, this sample also possessed antibacterial properties in the dark and its activity was two orders higher under UV/vis irradiation, which probably resulted from the different content of Cu^+^ in this sample (Table 2). Further study on the influence of properties of Cu deposits on antibacterial properties is currently being conducted. It is also possible that as in the case of bare OAP samples prepared with various durations of HT process (with antibacterial properties being correlated with their activity for oxidation of acetic acid (Fig. 11)), more efficient separation of charge carriers, and thus generation of ROS, is responsible for the best activity of Cu-modified OAPs (Fig. 9). In contrast to the study by Qiu et al. Cu/OAP did not show antibacterial activity under visible light irradiation, with only Ag-modified samples being able to inactivate bacteria. It should be pointed out that dark activity of Ag NPs participated in the resultant activity under visible light irradiation. However, activity under visible light was two orders higher than that in the dark. Under visible light irradiation, it is thought that Ag oxidation with simultaneous release of Ag cations (electron transfer from silver to the CB of titania under LSPR excitation) occurs. Therefore, free Ag cations could penetrate the cell membrane, resulting in the death of bacteria. Antibacterial tests for Ag sample prepared under aerobic conditions (Ag/OAP/O_2_) were also performed and the data are shown in Fig. 13 (bottom, right). Interestingly, the shape of curves in the dark and under irradiation for Ag/OAP/O_2_ was different from that for the Ag/OAP/Ar sample. The Ag/OAP/O_2_ sample was almost inactive during the first hour of irradiation and then acceleration of activity was observed after 2 h of irradiation. There are two possible reasons for this behavior: (1) type of bacteria and (2) properties of the photocatalyst. Benabbou et al. [100] and Bui et al. [101] suggested that bacteria inactivation proceeded in several steps due to the complex structure of the outer liposaccharide (LPS) membrane, which was not easily oxidized even by a photocatalytic process. However, in the present case, properties of the photocatalyst seem to be more crucial as activities of both Ag-modified samples differed significantly for the same tested bacteria. It is thought that an active form of the photocatalyst was formed during the first 2 h of irradiation. However, at present, it is difficult to determine whether more efficient release of Ag or better photoabsorption properties (broader LSPR) of this sample are responsible for the enhanced inactivation of bacteria.

**Fig. 14:**
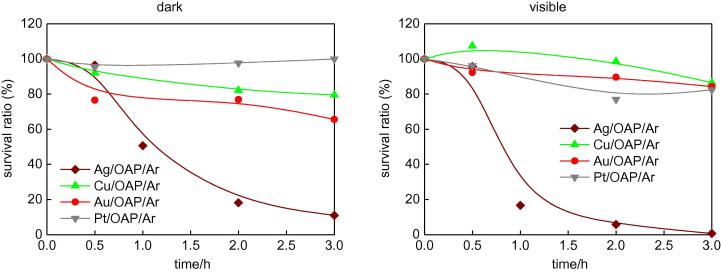
Antifungal (against *C. albicans*) activity of OAPs modified with metals under anaerobic conditions: (left) in the dark and (right) under vis irradiation (λ > 420 nm).

Antifungal properties in the dark and under visible irradiation were investigated, and the data obtained are shown in Fig. 14. As in the case of antibacterial properties, only Ag/OAP/Ar showed antifungal activities in the dark and under irradiation. These results were quite surprising since our preliminary study using commercial titania samples indicated stronger antifungal properties of Au-modified samples than those of Ag-modified samples under visible light irradiation (unpublished data). It is known that properties of metals differ significantly when deposited on various titania supports [49], and therefore different antimicrobial properties are obtained for different supports of metals. Interestingly, Ag/OAP/O_2_, which showed the strongest antibacterial properties under visible light irradiation (Fig. 13, bottom, right) was practically inactive against fungi, as shown in Fig. S13. It is likely that different mechanisms are responsible for inactivation of fungi and inactivation of bacteria, and therefore larger sizes and different shapes of Ag NPs prepared under aerobic conditions are not harmful for fungi. It is also probable that longer irradiation time is needed to activate the photocatalyst than in the case of bacteria suspension.

## 4 Conclusions

Modification of OAPs with noble metals results in significant enhancement of photocatalytic and antimicrobial activities. It has been found that the kind of metal and the method for preparation greatly influence the resultant properties and thus activities of photocatalysts. The surface of a photocatalyst prepared under aerobic conditions is enriched with oxygen, and thus enhanced photocatalytic activity for oxidative decomposition of acetic acid is achieved. Oxidation states of Pt and Au do not differ significantly depending on the method for preparation (mainly zero-charged), and only the surface of a photocatalyst prepared under aerobic conditions is enriched with hydroxyl groups. On the other hand, the surface of less noble metals is mainly positively charged. Although Cu is deposited as zero-valent NPs (violet color), it is rapidly oxidized in an ambient environment. In contrast, silver exists in both metallic (brown color of LSPR) and oxide forms.

Antibacterial properties of bare OAPs under UV/vis irradiation correlate with photocatalytic activities for decomposition of acetic acid and dehydrogenation of methanol, suggesting that the intrinsic properties of the photocatalyst are crucial, i.e., low rate of recombination of charge carriers on faceted anatase NPs with preferential distribution of shallow ETs rather than deep ETs. Enhanced antimicrobial properties of Ag/OAP/Ar under visible irradiation in comparison to dark activity indicate that plasmon resonance of Ag is responsible for photocatalyst activation. The highest antibacterial activity of Cu-modified samples under UV/vis irradiation correlate with the highest activity for acetic acid decomposition under UV/vis irradiation, suggesting prolongation of charge carriers’ lifetime by a possible heterojunction mechanism between two semiconductors, i.e., Cu_x_O/TiO_2_. Further study on the mechanism and on the best properties of photocatalysts is presently being conducted for possible application of metal-modified titania to environmental purification.

## Figures and Tables

**Table 1:** XRD analysis of metallic deposits in modified OAPs.

Code	Pt/OAP	Au/OAP	Cu/OAP			Ag/OAP		
Main component	Pt	Au		CuO			Ag_2_O	
Other components	–	–	Cu		Cu_2_O	Ag		AgO

*Deaerated samples*								
Crystallite size/nm	5.0	5.1	Cu	Cu_2_O	CuO	Ag	Ag_2_O	AgO
			1.8	10.9	4	n.d.	1.9	n.d

*Aerated samples*								
Crystallite size/nm	4.5	11.7	Cu	Cu_2_O	CuO	Ag	Ag_2_O	AgO
			4.1	n.d.	15.2	n.d.	9.4	n.d.

n.d. – not determined.

**Table 2:** Fraction of oxidation states of Pt, Au, Cu and Ag from deconvolution of XPS peaks of Pt 4f_7/2_, Au 4f_7/2_, Cu 2p_3/2_ and Ag 3d_5/2_.

Sample	Pt/OAP		Au/OAP			Cu/OAP		Ag/OAP		
Form	Pt(δ+)	Pt(0)	Au(*δ*+)	Au(0)	Au(*δ*-)	Cu^2+^	Cu^+^	Ag^2+^	Ag^+^	Ag(0)
*OAPs modified with metals under anaerobic conditions*										
Content (%)	39.1	60.9	6.4	90.3	3.3	23.9	76.1	0	92.4	7.6

*OAPs modified with metals under aerobic conditions*										
Content (%)	67.8	32.2	13.4	81.9	4.7	28.4	71.6	10.9	74.5	14.6
