# Supplementary material for: Noble metal-modified octahedral anatase titania particles with enhanced activity for decomposition of chemical and microbiological pollutants
Source: Chem Eng J. 2017 Jun 15;318:121–34. doi: 10.1016/j.cej.2016.05.138 (PMC5391806; doi:10.1016/j.cej.2016.05.138)
Supplement: Supplementary data 1 [file mmc1.pdf]

件名 EAAOP4_CHEMICAL ENGINEERING JOURNAL INVITATION LETTER

送信人 "EAAOP4" <eaaop4@easychair.org>

To "Zhishun Wei" <wei@cat.hokudai.ac.jp>

送信时间 2015年11月16日(一) 18:58:15

附件 Instructions ChemEngJ.doc

Dear Zhishun,

Your paper 270, Preparation of octahedral anatase titania particles for decomposition of chemical and microbiological pollutants has been selected to be considered for possible publication in Chemical Engineering Journal special issue on “Emerging Advanced Oxidation Processes for Disinfection and Micro-Pollutants Elimination”.

Please read the attached file for further instructions.

Best Regards,

Organizing Committee
