# Supplementary material for: Noble metal-modified octahedral anatase titania particles with enhanced activity for decomposition of chemical and microbiological pollutants
Source: Chem Eng J. 2017 Jun 15;318:121–34. doi: 10.1016/j.cej.2016.05.138 (PMC5391806; doi:10.1016/j.cej.2016.05.138)
Supplement: Supplementary data 2 [file mmc2.pdf]

Electronic supplementary information
for

Noble metal-modified octahedral anatase titania particles with enhanced activity for decomposition of chemical and microbiological pollutants

Z. Wei[a], M. Endo[a], K. Wang[a], E. Charbit[a], A. Markowska-Szczupak[b], B. Ohtani[a], E. Kowalska[a,*]

[a] Institute for Catalysis, Hokkaido University, N21, W10, 001-0021 Sapporo, Japan

[b] Institute of Chemical and Environmental Engineering, West Pomeranian University of Technology, ul. Pulaskiego 10, 70-322 Szczecin, Poland

*Corresponding author. Tel.: +81 117069130,

E-mail address: kowalska@cat.hokudai.ac.jp (E. Kowalska);

Table S1. XRD analysis of bare and modified OAPs

Samples	Crystalline size from 101 peak width/nm	Crystalline size from 004 peak width/nm	Aspect ratio
bare OAPs prepared for various durations of HT			
3hOAP	9.57	19.10	2.00
4.5hOAP	14.83	29.60	2.00
**6hOAP**	**17.23**	**33.00**	**1.92**
12hOAP	20.90	38.10	1.82
24hOAP	24.43	38.80	1.59
OAPs (**6hOAP**) modified with metals under anaerobic conditions			
Pt/OAP/Ar	17.44	33.50	1.92
Au/OAP/Ar	17.23	33.30	1.93
Cu/OAP/Ar	17.43	33.20	1.90
Ag/OAP/Ar	17.63	33.70	1.91
OAPs (**6hOAP**) modified with metals under aerobic conditions			
Pt/OAP/O_2	17.26	33.30	1.93
Au/OAP/O_2	17.03	30.50	1.79
Cu/OAP/O_2	17.21	34.00	1.98
Ag/OAP/O_2	17.25	32.90	1.91

DRS spectra of TNWs and products prepared from various HT durations:

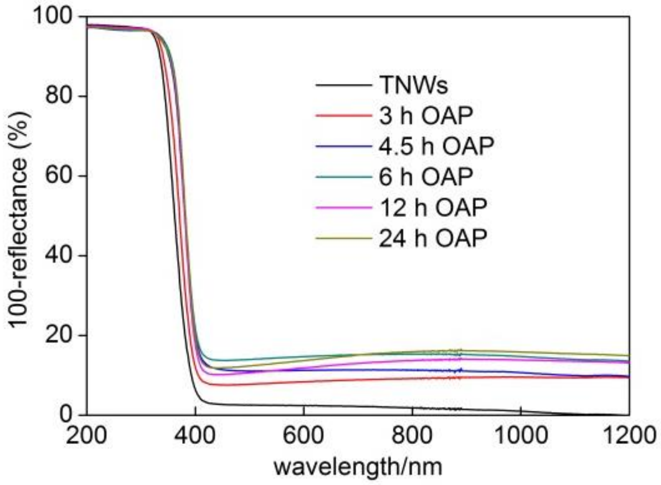

Fig. S1. DRS spectra of TNWs and bare OAP products prepared from different HT durations.

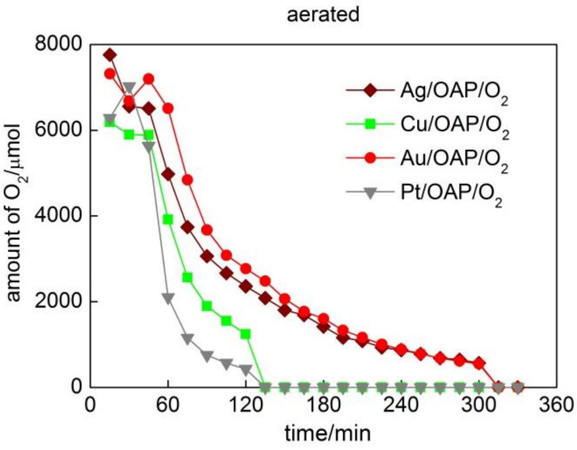

Fig. S2. Oxygen consumption during metal deposition under aerobic conditions.

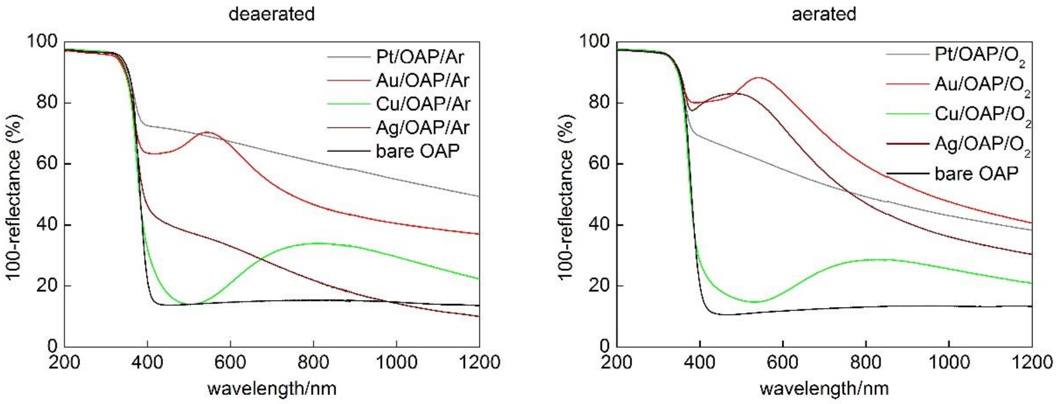

Fig. S3. DRS spectra of modified OAPs samples prepared under (left) anaerobic and (right) aerobic conditions; $BaSO_4$ used as reference.

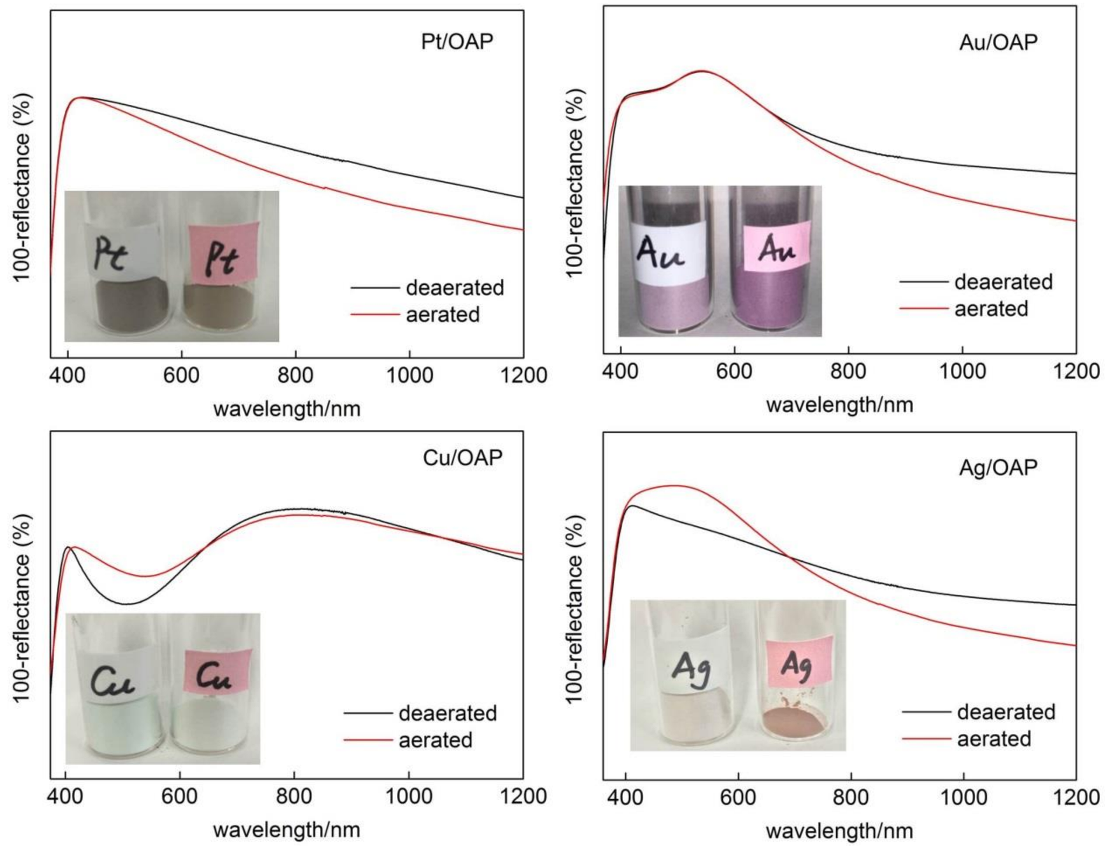

Fig. S4. Comparison of DRS spectra and photographs of modified OAPs samples prepared under anaerobic (left photographs) and aerobic (right photographs) conditions; bare OAPs used as reference.

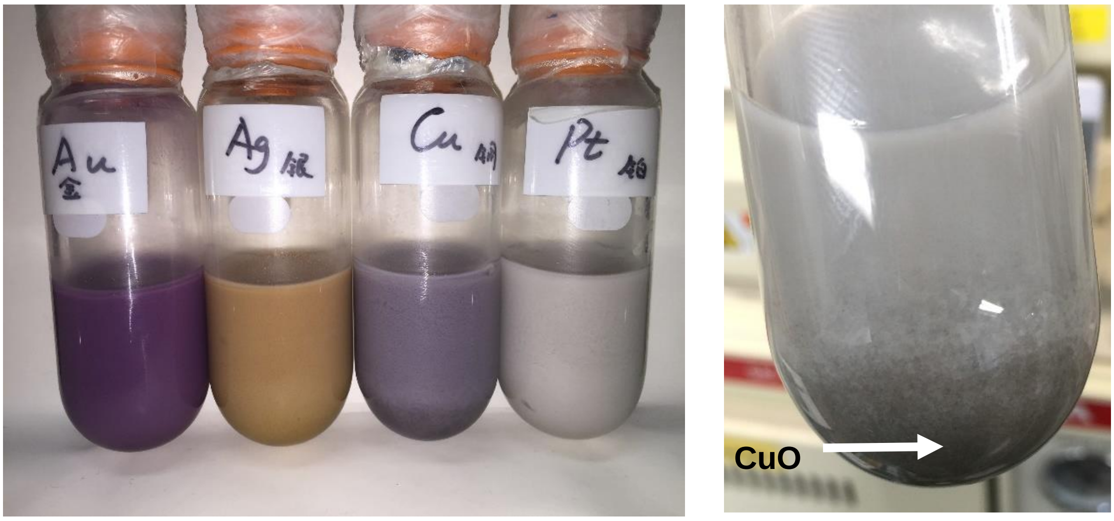

Fig. S5. Photographs of: (left) modified samples prepared under aerobic conditions, (right) formation of CuO during Cu photodeposition in the beginning of irradiation.

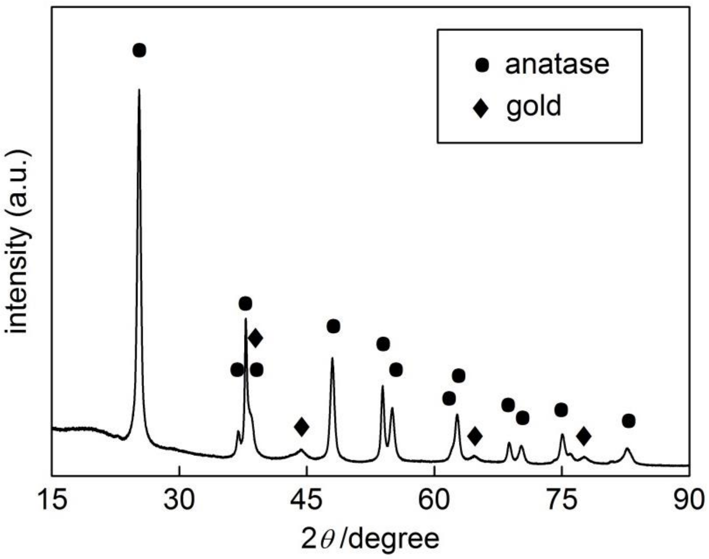

Fig. S6. XRD pattern for Au/OAPs prepared under aerobic conditions ● - anatase, ◆ - gold.

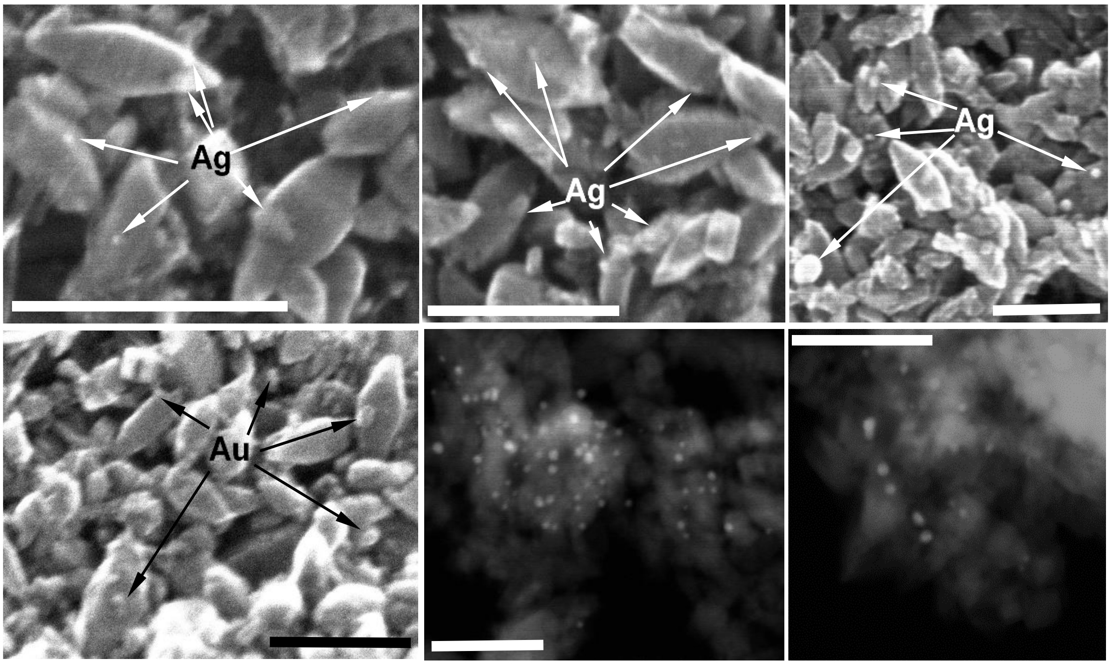

Fig. S7. STEM images of Ag (top) and Au (bottom) modified OAPs. Scale bars correspond to 80 nm.

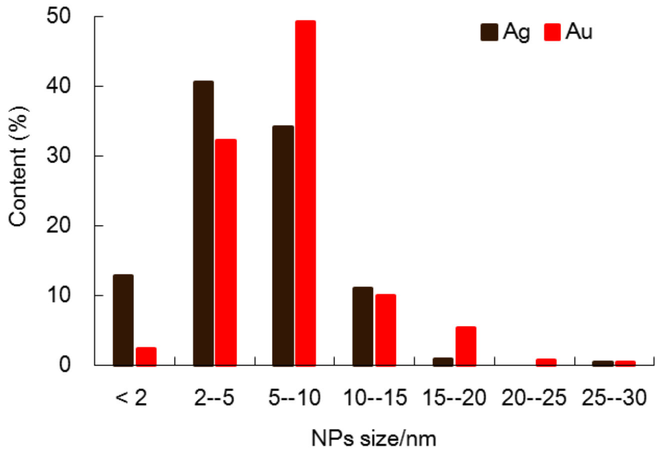

Fig. S8. Comparison of NPs sizes of Au and Ag deposited on OAPs under anaerobic conditions.

XPS results:

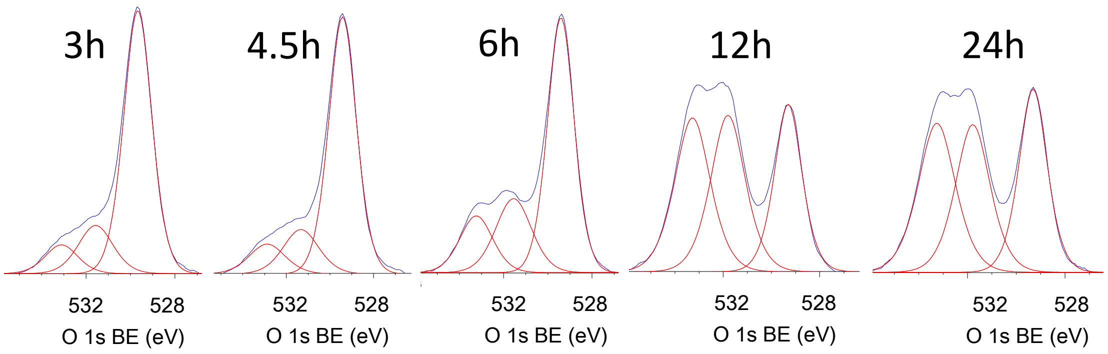

Fig. S9. XPS results for O 1s of bare OAP samples prepared at different duration of HT: (from left) 3, 4.5, 6, 12 and 24 h.

Table S2. XPS analysis of oxygen, titanium, carbon, noble metal of bare and modified OAPs

Samples	Content (at.%)				Ratio			Me^{a} (wt%)	Ti $2p_{3/2}$ (%)		O 1s (%)			C 1s (%)		
	Ti	O	C	Me^{a}	O/Ti	C/Ti	Me^{a}/Ti^{b}		Ti^{4+}	Ti^{3+}	TiO_2	Ti-OH[c]	Ti-OH[d]	C-C	C-OH	C=O
bare OAPs prepared for various durations of HT																
3hOAP	19.9	42.2	37.4	-	2.1	1.9	-	-	95.9	4.1	73.9	16.7	9.4	71.0	23.3	5.7
4.5hOAP	21.4	44.6	34.0	-	2.1	1.6	-	-	97.9	2.1	72.8	16.2	11.0	73.1	19.0	7.8
**6hOAP**	**15.0**	**37.7**	**47.3**	-	**2.5**	**3.2**	-	-	**97.3**	**2.7**	**60.1**	**23.3**	**16.6**	**67.0**	**23.9**	**9.1**
12hOAP	5.3	27.5	67.2	-	5.2	12.7	-	-	98.8	1.2	29.7	35.0	35.3	65.3	24.4	10.3
24hOAP	5.6	28.2	66.2	-	5.0	11.8	-	-	99.4	0.6	32.6	32.4	35.0	66.6	23.1	10.3
OAPs (**6hOAP**) modified with metals under anaerobic conditions																
Pt/OAP/Ar	19.7	41.2	38.8	0.29	2.1	2.0	1.47	3.5	98.5	1.5	70.8	19.6	9.6	65.1	27.7	7.2
Au/OAP/Ar	19.9	40.2	39.8	0.20	2.0	2.0	1.01	2.52	97.5	2.5	71.1	17.9	11.0	62.4	30.7	6.9
Cu/OAP/Ar	16.4	35.9	47.3	0.45	2.2	2.9	2.75	2.18	98.6	1.4	66.2	22.5	11.3	65.0	27.9	7.1
Ag/OAP/Ar	20.9	42.3	36.3	0.48	2.0	1.7	2.29	3.10	99.1	0.9	74.3	17.4	8.3	69.7	25.0	5.3
OAPs (**6hOAP**) modified with metals under aerobic conditions																
Pt/OAP/O_2	5.2	28.0	66.7	0.09	5.4	13.0	1.75	4.16	98.2	1.8	28.7	35.2	36.1	64.3	26.0	9.7
Au/OAP/O_2	3.2	24.0	72.7	0.06	7.45	22.6	1.86	4.66	97.9	2.1	19.9	41.4	38.7	60.3	30.2	9.5
Cu/OAP/O_2	12.9	34.2	52.7	0.21	2.7	4.1	1.63	1.29	99.4	0.6	54.7	32.1	13.2	58.0	35.1	6.9
Ag/OAP/O_2	15.2	38.8	45.9	0.16	2.6	3.0	1.05	1.42	98.3	1.7	59.8	25.2	15.0	77.1	13.9	9.0

[a]Me: metal, [b]Me/Ti: molar percent, [c]Ti-OH: Ti-(OH)-Ti/Ti_2O_3/C=O, [d]Ti-OH: Ti-OH/ C-OH.

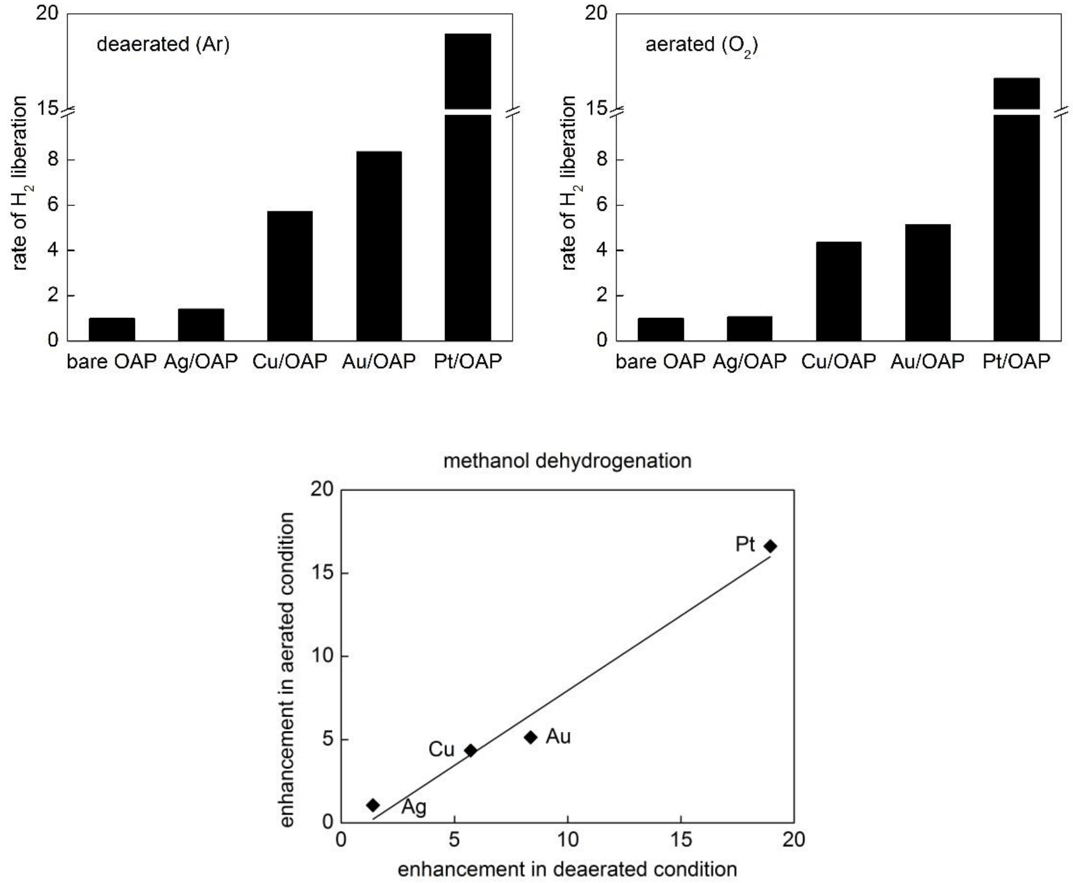

Fig. S10. (top) Methanol dehydrogenation under UV/vis irradiation on bare and modified OAPs prepared under (left) anaerobic and (right) aerobic conditions; (bottom) comparison of enhancement of hydrogen evolution after modification of OAPs with metals by two deposition methods.

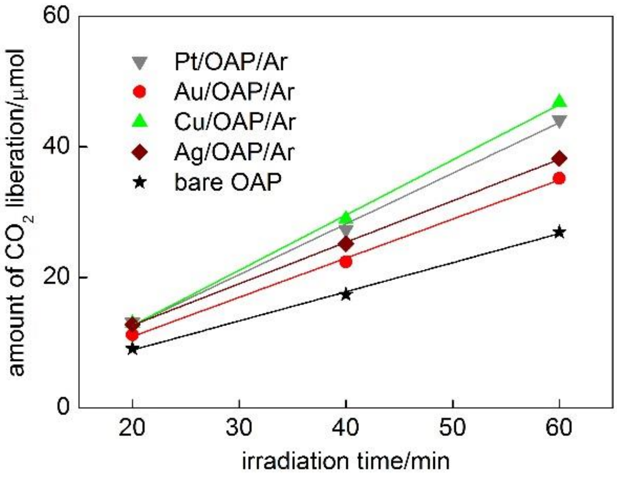

Fig. S11. Evolution of carbon dioxide during acetic acid decomposition under UV/vis irradiation on bare and modified OAPs prepared under anaerobic conditions.

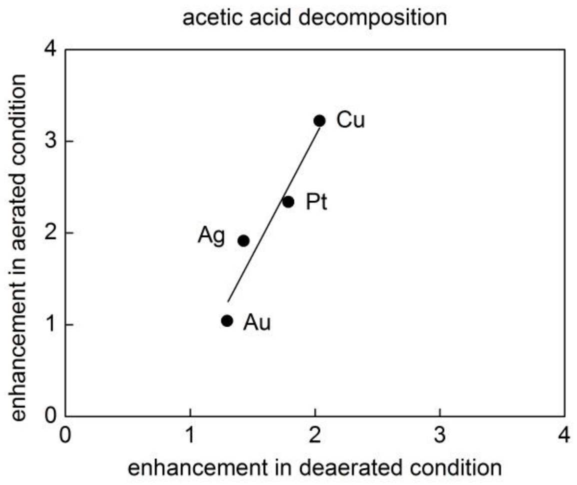

Fig. S12. Comparison of enhancement of acetic acid decomposition after modification of OAPs with metals by two deposition methods.

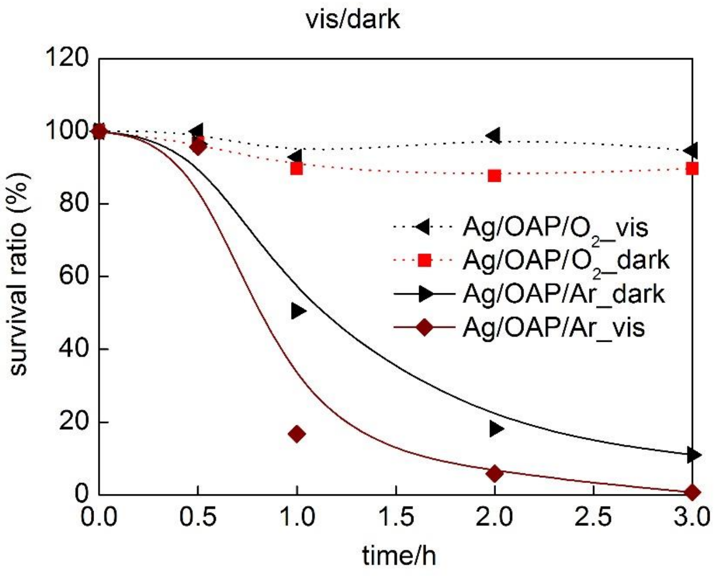

Fig. S13. Comparison of antifungal properties of Ag/OAP/Ar and Ag/OAP/O_2 samples.
